# Emergence of Carbapenem-Resistant Gram-Negative Isolates in Hospital Settings in Djibouti

**DOI:** 10.3390/antibiotics12071132

**Published:** 2023-06-30

**Authors:** Ayan Ali Ragueh, Mohamed Houmed Aboubaker, Sitani Idriss Mohamed, Jean-Marc Rolain, Seydina M. Diene

**Affiliations:** 1 Campus Balbala Croisement RN2-RN5, Université de Djibouti, Djibouti 1904, Djibouti; 2 MEPHI, IRD, AP-HM, IHU-Méditerranée Infection, Faculté de Pharmacie, Aix-Marseille Universite, 19-21 Boulevard Jean Moulin, CEDEX 05, 13385 Marseille, France; 3 Laboratoire de Biologie Médicale Mer-Rouge, Rue Moscou, Djibouti 1119, Djibouti; houmedm@yahoo.fr; 4 Laboratoire de Biologie et de Biochimie Clinique de L’hôpital Général Peltier, 1323, Avenue Maréchal, Djibouti 1119, Djibouti

**Keywords:** Gram-negative bacilli, carbapenem resistance, Djibouti

## Abstract

**Introduction**: The antimicrobial resistance (AMR) of bacteria is increasing rapidly against all classes of antibiotics, with the increasing detection of carbapenem-resistant isolates. However, while growing prevalence has been reported around the world, data on the prevalence of carbapenem resistance in developing countries are fairly limited. In this study, we investigated and determined the resistance rate to carbapenems among multidrug-resistant Gram-negative bacteria (MDR-GNB) isolated in Djibouti and characterized their resistance mechanisms. **Results**: Of the 256 isolates, 235 (91.8%) were identified as Gram-negative bacteria (GNB). Of these GNBs, 225 (95.7%) isolates exhibited a multidrug resistance phenotype, and 20 (8.5%) isolates were resistant to carbapenems, including 13 *Escherichia* coli, 4 *Acinetobacter baumannii*, 2 *Klebsiella pneumoniae* and 1 *Proteus mirabilis*. The most predominant GNB in this hospital setting were *E. coli* and *K. pneumoniae* species. Carbapenemase genes such as *bla*_OXA-48_ and *bla*_NDM-5_ were identified, respectively, in six and four *E. coli* isolates, whereas the carbapenemase *bla*_NDM-1_ was identified in three *E. coli*, two *K. pneumoniae*, one *P. mirabilis* and one *A. baumannii*. Moreover, three *A. baumannii* isolates co-hosted *bla*_OXA-23_ and *bla*_NDM-1_. **Materials and Methods**: A total of 256 clinical strains collected between 2019 and 2020 were identified using matrix-assisted laser desorption/ionization time-of-flight (MALDI-TOF). Antibiotic susceptibility testing was performed using disk diffusion and E-test methods. Real-time polymerase chain reaction (RT-PCR), standard PCR and sequencing were used to investigate genes encoding for extended-spectrum-β-lactamases, carbapenemases and colistin resistance genes. **Conclusions**: We report, for the first time, the presence of MDR-GNB clinical isolates and the emergence of carbapenem-resistant isolates in Djibouti. In addition to performing antimicrobial susceptibility testing, we recommend phenotypic and molecular screening to track the spread of carbapenemase genes among clinical GNB isolates.

## 1. Introduction

The emergence and rapid evolution of antimicrobial resistance (AMR) represents a global challenge for the control of infectious diseases [1]. The rise of multidrug resistance has been observed in all bacteria, including clinically important Gram-negative bacilli (GNB), and is now a serious challenge encountered by healthcare professionals [2]. These GNB are responsible for a variety of community- and healthcare-acquired infections, and their resistance is mainly mediated by extended spectrum-β-lactamases (ESBLs) and carbapenemases [3]. The increasing prevalence worldwide of carbapenem-resistant *Enterobacteriaceae* (CRE) has compromised carbapenem-based treatments which are considered to be antibiotics of last resort or “last-line agents” in hospitals and long-term care facilities [4,5]. Since the first description of carbapenemase-producing *Enterobacteriaceae* (NmcA) in 1993, a large variety of carbapenemases have been identified, belonging to three molecular β-lactamase classes, including the Ambler class A (KPC, IMI and GES), class B (NDM, IMP and VIM) and class D (OXA-48, OXA-23, OXA-24 and OXA-58) [6]. They have emerged in and spread to different parts of the world, including Mediterranean countries, in recent years [7,8]. Among the newly emerging β-lactamases, the dramatic global spread of NDM remains one of the most worrying antibiotic resistance events caused by metallo-β-lactamase (MBL). It was first reported from carbapenem-resistant *K. pneumoniae* recovered from a Swedish patient previously hospitalized in India [9].

Given their ability to hydrolyze most β-lactams (with the exception of monobactams), including the carbapenems, NDM-producing isolates responsible for infections are very difficult to treat [10]. These NDM producers include mainly *Enterobacteriaceae*, *Acinetobacter spp*. and, more rarely, *Pseudomonas aeruginosa* isolates, usually responsible for severe nosocomial infections, including urinary tract infections, peritonitis, septicemia and pulmonary infections. As reported in the literature, the Indian subcontinent, the Balkans region and the Middle East are considered to be the main reservoirs of NDM producers [11]. First identified from *K. pneumoniae* in Turkey in 2001, *bla*_OXA-48_ has also been extensively reported as a source of nosocomial infection outbreaks in many parts of the world, notably in countries in the Mediterranean region [12].

In Africa, data on the distribution and prevalence of carbapenem resistance among MDR-GNB isolates are still limited. In Djibouti, to the best of our knowledge, no study on the resistance rate or molecular epidemiology of carbapenem-resistant GNBs has been described so far. Here, we investigate the occurrence of carbapenem-resistant GNB isolates from hospitalized patients and outpatients in Djibouti and then characterize their molecular resistance mechanisms. 

## 2. Results

### 2.1. Sample Culture

**Figure 1:**
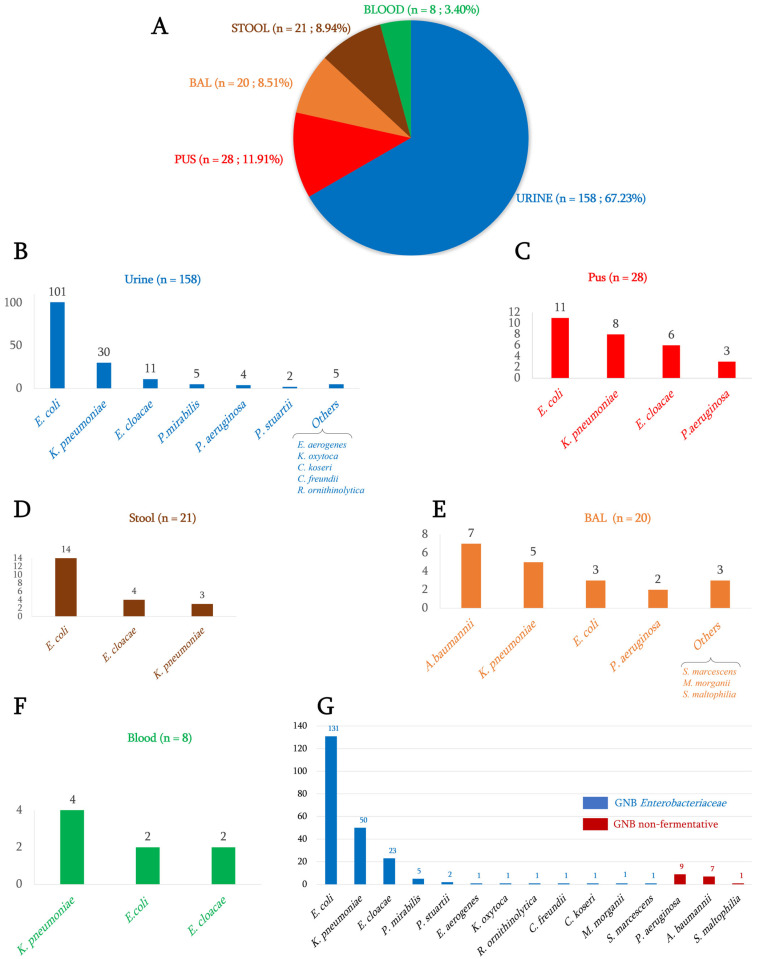
Distribution of clinical GNB isolates by collected samples. (**A**): number and percentage of bacterial isolates per type of sample; (**B**): distribution of bacterial species isolated from urine; (**C**): from pus; (**D**): from stool; (**E**): from bronchoalveolar lavage; (**F**): from blood; (**G**): distribution of all bacterial isolates analyzed in this study.

The 235 isolates collected between 2019 and 2020 at the Mer-Rouge laboratory in Djibouti were from clinical samples of hospitalized patients including urine (n = 158; 67.3%), pus (n = 28; 11.9%), stool (n = 21; 8.9%), Bronchoalveolar lavage (BAL) (n = 20; 8.5%) and blood (n = 8; 3.4%) (Figure 1A).

Among the GNB, *Enterobacteriaceae* represented 92.8% (n = 218), and non-fermenting GNB were 7.2% (n = 17). Of the 218 *Enterobacteriaceae,* 60.1% (n = 131) were *Escherichia coli*, which represents the most predominant species, followed by *K. pneumoniae* (22.9%, n = 50) and *Enterobacter cloacae* (10.6%; n = 23). A total of 2.3% (n = 5) of the isolates were *Proteus mirabilis* and 0.9% (n = 2) were *Proteus stuartii*. In addition, a single isolate of each of *Citrobacter freundii*, *Citrobacter koseri*, *Enterobacter aerogenes*, *Klebsiella oxytoca*, *Morganella morganii*, *Raoultella ornithinolytica* and *Serratia marcescens* was identified. Among the 17 non-fermenting GNB, 52.9% (n = 9) were *P. aeruginosa*, 41.2% (n = 7) were *Acinetobacter baumannii* and 5.9% (n = 1) was *S. maltophilia* (Figure 1B).

### 2.2. Antibiotic Susceptibility Test (AST)

**Figure 2:**
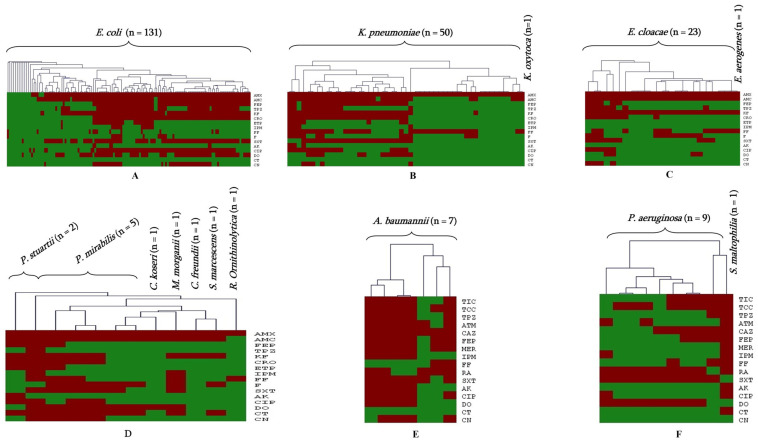
Antimicrobial susceptibility profile of Enterobacteriaceae and non-fermenting GNB: (**A**) *E. coli*; (**B**) *K. pneumoniae* and *K. oxytoca*; (**C**) *E. cloacae* and *E. aerogenes;* (**D**) *P. stuartii*, *P. mirabilis*, *C. koseri*, *C. freundii*, *M. morganii*, *S. marcescens* and *R. ornithinolytica*; (**E**) *A. baumannii;* and (**F**) *P. aeruginosa* and *S. maltophilia*. AMX = Amoxicilline, AMC = Amoxicilline/clavulanic acid, FEP = Cefepime, TPZ = Piperacillin + Tazobactam, KF = Cephalothin, TIC = Ticarcillin, TCC = Ticarcillin/Clavulanic acid, CRO = Ceftriaxone, CAZ = Ceftazidime, ETP = ertapenem, MER = Meropenem, IPM = imipenem, ATM = Aztreoname, FF = Fosfomycin, F = Nitrofurantoïne, SXT = Trimethoprim/sulfamethoxazol, AK = Amikacin, CIP = Ciprofloxacine, DO = Doxycycline, CT = colistin and CN = Gentamicin, RA = Rifampicin. Green color refers to susceptibility to the antibiotic and red color refers to resistant ones.

As shown in Figure 2, the AST revealed the phenotypic resistance among the 235 GNB isolates. Indeed, 95.7% (n = 225) exhibited a multidrug-resistant profile (MDR), and 135 (57.4%) isolates were resistant to third-generation cephalosporins (3GCs), specifically to ceftriaxone (CRO). Resistance to ceftriaxone was observed mostly in *E. coli* and *K. pneumoniae*, showing significant rates of resistance of 36.2% (n = 85) and 11.1% (n = 26), respectively.

As presented in Table 1 and Table 2, resistance to carbapenems (i.e., ertapenem and/or imipenem) was confirmed using the E-test method and was observed in 20 (8.2%) of the MDR isolates, including 13 *E. coli* (65%), 4 *A. baumannii* (20%), 2 *K. pneumoniae* (10%) and 1 *P. mirabilis* (5%). No colistin resistance was observed in our collection, except in those that are naturally colistin-resistant, like *P. mirabilis*. 

### 2.3. Prevalence of ESBL and Carbapenemase Genes

In total, 204 of the 235 (86.8%) MDR-GNB isolates were positive for at least one of the investigated ESBL genes. Of the ESBL genes commonly reported in human medicine, the *bla*_CTX-M_ gene was harbored by the majority of the isolates (n = 168/204; 82.3%), followed by *bla*_TEM_ (n = 115/204; 56.3%) and *bla*_SHV_ (n = 81/204; 39.7%). The presence of the *bla*_CTX-M_ gene was predominant in *E. coli* (n = 99/168; 58.9%), *K. pneumoniae* (n = 62/168; 36.9%) and *E. cloacae* (n = 7/168; 4.1%). However, as presented in Table 3, several of the isolates contained at least two or more ESBL genes (n = 126/204; 61.7%). Regarding carbapenemase genes, the RT-PCR results showed that the *bla*_NDM_ variants were the most predominant genes detected in 13 isolates (65%), followed by the *bla*_OXA-48_ variant (*bla*_OXA-181_ here, identified after standard PCR and sequencing) in 6 isolates (30%) and *bla*_OXA-23_ in 4 isolates (20%). Interestingly, as presented in Table 2, the pandemic class D carbapenemase OXA-23 enzyme was detected in four out of the seven isolated *A. baumannii* strains from broncho-alveolar lavage samples, and three of them also harbored the metallo-β-lactamase NMD-1 enzyme.

## 3. Discussion

Several studies have shown that AMR is a problem in sub-Saharan Africa, and the available data show that multidrug resistance is widespread in Gram-negative bacteria [13,14,15]. Our study of clinical samples in Djibouti revealed rather alarming rates of drug resistance among Gram-negative organisms (95.7%). We found that almost 92% of infections were due to Gram-negative isolates and that approximately 58% of them were resistant to third-generation cephalosporins (3GCs). Our results revealed that *E. coli* had a higher prevalence of resistance to 3GCs: 36.2% presented resistance to ceftriaxone and therefore had a high phenotypic resistance profile to cephalosporins. Other studies in East Africa have shown similar rates, as is the case in Tanzania, where 30% of *E. coli* were resistant to ceftriaxone [16], or lower rates, as is the case in Kenya and Uganda at 12.8% and 2.9%, respectively [17,18]. This remains a concerning finding among the poor populations in the Horn of Africa. Similar results in different regions of the world have been observed, as previously reported [19]. Our findings indicate that the majority of ESBL-producer isolates harbored the *bla*_CTX-M_ gene, followed by *bla*_TEM_ and *bla*_SHV_. Studies in Saudi Arabia and Palestine have also reported high prevalence rates (93.5% and 100%, respectively) of *bla*_CTX-M_ genes in ESBL-producing *Enterobacteriaceae* isolates [20,21]. These results remain in agreement with several other studies because these CTX-M enzymes have become predominant ESBLs in many African countries [22]. Our findings suggest a significant spread of *bla*_CTX-M_-positive *Enterobacteriaceae* circulating in our environment, which continues to cause community and nosocomial infections. For the first time, in this study, we describe 20 (8.5%) species of multidrug-resistant Gram-negative bacteria carrying carbapenemases in Djibouti, especially *bla*_NDM-1_, *bla*_NDM-5_, *bla*_OXA-23_ and *bla*_OXA-181_.This prevalence is lower compared to that found in studies carried out in Tanzania, Sudan and Morocco, which found 35.24%, 45% and 85.5% prevalence rates, respectively [12,23,24]. The most frequently identified carbapenemase genes in Djibouti were *bla*_NDM_ followed by *bla*_OXA-48_. This result is in agreement with the conclusions of studies carried out in Qatar [25] and in many countries in the Arabian Peninsula which confirmed that currently, the NDM and OXA-48 enzymes are the two main carbapenemases [26,27,28]. All positive RT-PCRs targeting the *bla*_OXA-48_ gene were identified as *bla*_OXA-181_ via standard PCR and sequencing. The OXA-181 protein differs from OXA-48 by four amino acids and, like the NDM-1 enzyme, its origin is epidemiologically linked to southern Asia, most often the Indian subcontinent [29,30]. Given the strong presence of people originating from the Asian continent and the Indian subcontinent, the existence of strains carrying *bla*_NDM_ and *bla*_OXA-48_ genes and circulating in Djibouti is not surprising. We also detected three isolates of *A. baumannii* co-hosting the *bla*_OXA-23_ gene and the *bla*_NDM-1_ gene. This finding is concerning since the spread of such MDR pathogens in hospital settings may compromise β-lactam-based treatments. Although β-lactams are the most commonly used antibiotics in community and hospital settings in Djibouti, and often inappropriately so, carbapenems are beginning to be prescribed as a treatment of last resort for life-threatening infections caused by multidrug-resistant bacteria. Other studies first carried out in East Africa reported the presence of the *bla*_NDM_ gene in Gram-negative bacteria, including one strain of *A. baumannii* in Kenya in 2013 [31,32], three *bla*_NDM_ genes in Ethiopia in 2017 [31], and in surrounding countries like Yemen in which the *bla*_NDM_ gene has also been reported in 2014 [33]. The emergence of these species exhibiting an MDR phenotype can be explained by the importation of resistant isolates from countries bordering Djibouti during travel or migratory flows. 

## 4. Materials and Methods

This was a cross-sectional study conducted at the Mer-Rouge laboratory in Djibouti, involving clinical isolates collected between January 2019 and July 2020. These strains were isolated from urine, pus, blood and respiratory samples. They were then returned to Tryptic Soy Agar (TSA), Columbia Blood Agar (COS) and MacConkey agar and incubated for 24 h at 37 °C. After overnight incubation at 37 °C, colonies showing different morphologies were picked up from each selective plate and tested separately via MALDI-TOF MS for identification, using the Microflex LT spectrometer (Bruker Daltonics, Bremen, Germany) [34,35]. Antibiotic susceptibility testing was performed using the Mueller–Hinton agar disc diffusion method (Fluka, St. Louis, MO, USA) and interpreted according to the recommendations of the European Committee for Antimicrobial Susceptibility Studies (EUCAST: www.eucast.org, accessed on 15 June 2022) [36]. Two different panels of 16 antibiotic disks (Bio-Rad, Gémenos, France) were used for fermenting GNB and non-fermenting GNB, respectively. The ESBL profile was detected by observing a champagne cork between third- or fourth-generation cephalosporin and clavulanic acid. The minimum inhibitory concentrations (MICs) of ertapenem and imipenem were determined using the E-test method (bioMérieux, La Balmes-les-Grottes, France). Carbapenemase activity was screened for using the ß-CARBA NP test (Bio-Rad, Hercules, CA, USA) [37]. The automatic robot EZ1 (Qiagen BioRobot EZ1, Hilden, Germany) was used to extract bacterial DNA with the extraction kit EZ1 DNA (Qiagen, Hilden, Germany) according to the manufacturer’s guidelines. The presence of carbapenemase genes, including *bla*_KPC_, *bla*_VIM_, *bla*_NDM_, *bla*_OXA-23_, *bla*_OXA-24_, *bla*_OXA-48_ and *bla*_OXA-58_, was checked via real-time PCR (C1000 Thermal Cycler, Bio-Rad, USA) using primers and probes previously described [38,39], while ESBL-encoding genes (*bla*_CTX-M_, *bla*_TEM_ and *bla*_SHV_) were investigated via standard PCR using previously reported primers [40], and positive PCR products were sequenced using BigDye Terminator chemistry. The sequences were then identified via BLAST against the ARG-ANNOT [41] and NCBI databases. 

## 5. Conclusions

For the first time, here, we reported the prevalence of genes conferring resistance to carbapenems in several Gram-negative bacteria circulating in Djibouti. In addition, these results could serve as a basis for future studies and for the assessment of trends in infections caused by MDR-GNB. The highlighted emergence of carbapenem-resistant isolates, such as *A. baumannii* co-hosting two carbapenemases, is of concern. There is, therefore, an urgent need to establish routine surveillance systems for carbapenem resistance among MDR-GNBs to prevent the spread of such pathogens in the country.

## Figures and Tables

**Table 1:** Antimicrobial susceptibility profile of carbapenem-resistant *Enterobacteriaceae* isolates and carbapenemase-producing genes.

Strains	Years	AMX	AMC	FEP	TPZ	CRO	ETP	IPM	FF	F	SXT	AK	CIP	DO	CT	CN	Carbapenemase Genes
*E. coli*	2019	R	R	R	R	R	R	R	S	R	R	S	R	R	S	R	*bla*_NDM-1_
*E. coli*	2019	R	R	R	R	R	R	R	S	S	R	S	R	S	S	S	*bla*_NDM-1_
*E. coli*	2020	R	R	R	R	R	R	R	S	S	R	S	R	S	S	S	*bla*_NDM-5_
*E. coli*	2020	R	R	R	R	R	R	R	S	R	R	S	R	R	S	S	*bla*_NDM-5_
*E. coli*	2020	R	R	R	R	R	R	R	S	R	R	S	R	R	S	R	*bla*_NDM-5_
*E. coli*	2020	R	R	R	R	R	R	S	S	S	S	S	R	R	S	S	*bla*_NDM-5_
*E. coli*	2020	R	R	R	R	R	R	R	S	S	S	S	R	S	S	S	*bla*_NDM-1_
*E. coli*	2020	R	R	R	R	R	R	S	S	S	R	S	R	R	S	R	*bla*_OXA-181_
*E. coli*	2020	R	R	R	R	R	R	R	R	S	R	S	R	R	S	S	*bla*_OXA-181_
*E. coli*	2020	R	R	R	R	R	R	R	S	S	R	S	R	R	S	S	*bla*_OXA-181_
*E. coli*	2020	R	R	R	R	R	R	S	S	S	R	S	R	R	S	R	*bla*_OXA-181_
*E. coli*	2020	R	R	R	R	R	R	R	S	S	R	S	R	R	S	S	*bla*_OXA-181_
*E. coli*	2020	R	R	R	R	R	R	R	S	S	R	S	R	R	S	S	*bla*_OXA-181_
*K. pneumoniae*	2020	R	R	R	R	R	R	R	R	R	S	R	R	S	S	S	*bla*_NDM-1_
*K. pneumoniae*	2020	R	R	R	R	R	R	R	R	R	S	R	R	S	S	S	*bla*_NDM-1_
*P. mirabilis*	2020	R	R	R	R	R	R	R	R	S	R	S	R	R	R	S	*bla*_NDM-1_

**Table 2:** Antimicrobial susceptibility profile of non-fermenting bacteria harboring OXA-23 and/or NDM-1 carbapenemase enzymes.

Scheme	Years	TIC	TCC	TPZ	ATM	CAZ	FEP	MER	IMP	FF	RA	SXT	AK	CIP	DO	CT	CN	Carbapenemase Genes
*A. baumannii*	2020	R	R	R	R	R	R	R	R	R	R	S	S	S	S	S	S	*bla*_OXA-23_
*A. baumannii*	2020	R	R	R	R	R	R	R	R	S	R	R	R	R	S	S	R	*bla*_OXA-23_, *bla*_NDM-1_
*A. baumannii*	2020	R	R	R	R	R	R	R	R	S	R	R	R	R	S	S	R	*bla*_OXA-23_, *bla*_NDM-1_
*A. baumannii*	2020	R	R	R	R	R	R	R	R	S	R	R	R	R	S	S	R	*bla*_OXA-23_, *bla*_NDM-1_

R = resistance, S = Sensitive. AMX = Amoxicilline, AMC = Amoxicilline/clavulanic acid, FEP = Cefepime, TPZ = Piperacillin + Tazobactam, KF = Cephalothin, TIC = Ticarcillin, TCC = Ticarcillin/Clavulanic acid, CRO = Ceftriaxone, CAZ = Ceftazidime, ETP = ertapenem, MER = Meropenem, IPM = imipenem, ATM = Aztreoname, FF = Fosfomycin, F = Nitrofurantoïne, SXT = Trimethoprim/sulfamethoxazol, AK = Amikacin, CIP = Ciprofloxacine, DO = Doxycycline, CT = colistin and CN = Gentamicin, RA = Rifampicin, IMP = imipenem.

**Table 3:** Distribution of ESBL and carbapenemase-producing isolates.

	*E. coli*	*K. pneumoniae*	*E. cloacae*	*A. baumannii*	*P. mirabilis*	Total (n = 228)
Single ESBL genes						
***bla*_CTX-M_**	38	1	3	0	0	42 (18.7%)
***bla*_TEM_**	23	0	2	0	0	25 (11.6%)
***bla*_SHV_**	0	14	1	0	0	15 (6.7%)
**Total**	**61**	**15**	**6**	**0**	**0**	**82 (36.6%)**
ESBL gene combinations						
***bla*_CTX-M_ + *bla*_TEM_**	36	20	4	0	0	60 (26.7%)
***bla*_CTX-M_ + *bla*_SHV_**	13	23	0	0	0	36 (16.1%)
***bla*_CTX-M_ + *bla*_TEM_ + *bla*_SHV_**	12	18	0	0	0	30 (13.4%)
**Total**	**61**	**61**	**4**	**0**	**0**	**126 (56.2%)**
**Carbapenemase** genes						
***bla*** **_NDM-1_**	**3**	2	0	0	1	6 (2.7%)
***bla*** **_NDM-5_**	**4**	0	0	0	0	4 (1.8%)
***bla*** **_OXA-181_**	**6**	0	0	0	0	6 (2.7%)
***bla*** **_OXA-23_**	**0**	0	0	1	0	1 (0.4%)
***bla*** **_OXA-23_ +** ***bla*** **_NDM-1_**	**0**	0	0	3	0	3 (1.4%)
**Total**	**13**	**2**	**0**	**4**	**1**	**20 (8.9%)**

## Data Availability

Not applicable.
